# Differences in the diagnosis and treatment decisions for children in care compared to their peers: An experimental study on post‐traumatic stress disorder

**DOI:** 10.1111/bjc.12379

**Published:** 2022-06-14

**Authors:** Rosie McGuire, Sarah L. Halligan, Richard Meiser‐Stedman, Lucy Durbin, Rachel M. Hiller

**Affiliations:** ^1^ Department of Psychology University of Bath Bath UK; ^2^ Department of Psychiatry University of Cape Town Cape Town South Africa; ^3^ Department of Clinical Psychology & Psychological Therapies, Norwich Medical School University of East Anglia Norwich UK; ^4^ Division of Psychology and Language Sciences University College London London UK; ^5^ Anna Freud National Centre for Children and Families London UK

**Keywords:** mental health, PTSD, trauma

## Abstract

**Objectives:**

Despite evidence of high rates of diagnosable mental health difficulties in children in care, there remains ongoing debate around the appropriateness of traditional diagnoses and treatments. The aim of this study was to quantitatively explore whether mental health diagnosis and treatment decision‐making differed when a young person was identified as being in care, specifically focused on post‐traumatic stress disorder (PTSD). PTSD is a trauma‐specific mental health disorder with rates substantially higher in children in care versus their peers.

**Methods:**

Participants were 270 UK mental health professionals who completed an online survey. Participants were randomized to receive one of two vignettes, which were identical in their description of a teenage boy experiencing PTSD symptoms, except in one he was in foster care and in the other he lived with his mother. Participants were asked to select a primary diagnosis, treatment approach, and potential secondary diagnosis.

**Results:**

Professionals were twice as likely to choose a primary diagnosis of PTSD and a National Institute for Clinical Excellence (NICE)‐recommended PTSD treatment when randomized to the mother vignette versus the foster carer vignette. Selecting PTSD as the primary diagnosis made clinicians three times more likely to select a NICE‐recommended treatment for PTSD. Developmental trauma was the most common ‘diagnosis’ for both groups, although this led to different treatment decisions.

**Conclusions:**

In the context of PTSD, we found children in care face diagnosis and treatment decision‐making biases. Practice implications are discussed.

Practitioner points

- When given identical information, mental health professionals were less likely to diagnose a child in care (vs. a child not in care) with PTSD.
- Related to the lesser detection of PTSD, mental health professionals were less likely to choose NICE‐recommended PTSD treatments for a child in care.
- Overall, if PTSD was identified, the professional was three times more likely to choose a NICE‐recommended PTSD treatment, yet many still did not.
- Developmental trauma was the most common diagnosis or presenting issue selected, regardless of whether or not the child was in care.

## INTRODUCTION

In the United Kingdom (UK) there are over 90,000 young people who have been removed from the care of their biological parent(s) and placed in the care of a local authority. Most have been moved into care due to abuse or neglect and all have experienced significant adversity and upheaval (Department for Education, 2018). Once in care, instability can continue, including multiple placement changes and seperation from siblings (Department for Education, 2018). High rates of mental health difficulties have been well‐documented in this group (e.g., Blower et al., 2004; Ford et al., 2007). Nevertheless, the literature continues to show that the mental health needs of children in care often remain unmet, with reported struggles in accessing appropriate support via child and adolescent mental health services (CAMHS), and reported dissatisfaction with how needs are understood and addressed (e.g., Hiller et al., 2020; Larsen et al., 2018; Minnis et al., 2006). However, it is also the case that many young people, whether in care or not, can struggle to access our stretched mental health services (NHS England, 2015). It remains unclear whether this is indeed an issue unique to, or worse for, children in care or whether this reflects the general state of child mental health services. The goal of this study was to quantitatively explore diagnosis and treatment decisions for children in care versus those not in care, specifically focusing on post‐traumatic stress disorder (PTSD).

PTSD is a trauma‐specific mental health disorder. Rates of PTSD in children in care are 12 times higher than in their peers, with evidence of particularly high rates for those on waiting lists for mental health services (Ford et al., 2007; Morris et al., 2015). If left unaddressed, PTSD can become a chronic and debilitating mental health difficulty, so it is important that clinicians are able to identify the disorder and provide appropriate support. However, evidence from the United States and UK suggests PTSD is often poorly detected in children within child welfare settings (Grasso et al., 2009; Miele & O'Brien, 2010; Morris et al., 2015). This may be partially due to ongoing clinical and academic debate around whether diagnostic frameworks are applicable to children in care (Teicher et al., 2022), despite evidence to the contrary (e.g., Ford et al., 2007). While there is some evidence that PTSD is under‐detected in this group of young people, it remains unclear whether the under or misdiagnosis of PTSD is uniquely problematic for those in care, and what implications this may have on treatment decisions.

A diagnostic‐framework driven formulation can be particularly useful to highlight the most appropriate evidence‐based treatment options. For young people with PTSD, the National Institute for Clinical Excellence (NICE) suggest that trauma‐focused cognitive behavioural therapy (tf‐CBT) is the first‐line best‐evidenced treatment for PTSD, with eye‐movement desensitization and reprocessing (EMDR) the next recommended treatment if they do not respond to tf‐CBT (NICE, 2018). Alongside debate about the use of diagnoses, there has also been much clinical and academic debate about the appropriateness of cognitive‐behavioural approaches for young people who have experienced more complex trauma (sometimes called ‘developmental trauma’; DeJong, 2010; van der Kolk, 2017). Yet, multiple reviews show that tf‐CBT is an effective treatment for children who have experienced ongoing maltreatment, which includes a smaller set of studies specifically with children in foster care (e.g., Bastien et al., 2020; Bennett et al., 2021).

Young people may face various barriers when trying to access child mental health services and accessing assessments and evidence‐based treatments – including those driven by underfunding of these systems (Golding, 2010). A review of barriers to the use of PTSD treatments specifically, found a lack of access to staff training and supervision were important issues (Finch et al., 2020). Clinician beliefs were also important, which included concerns about the emotional burden of discussing trauma, less favourable attitudes towards evidence‐based interventions, and a more psychodynamic or humanistic therapeutic orientation (Finch et al., 2020). Many of these barriers may particularly apply to children in care, where anecdotal and qualitative evidence suggests a reluctance to use diagnostic labels and NICE‐recommended treatments, and where decision‐making in the face of complex comorbidities and crises is commonplace (Callaghan et al., 2003; Minnis & Del Priore, 2001).

In this study, we sought to quantitatively explore whether a child being in care changed diagnosis and treatment decision‐making, specifically related to PTSD. UK‐based child and adolescent mental health care providers were randomized to receive one of two vignettes, which were identical except for one describing the young person as being in care. The primary aim of this study was to explore whether a young person being identified as in care (vs. not being in care) was associated with differences in (i) the identification of PTSD and (ii) the use of a NICE‐recommended PTSD treatment (tf‐CBT or EMDR). As part of this aim, we also sought to describe broader diagnosis and treatment decision‐making, to understand which diagnoses were selected if not PTSD, and what this meant for subsequent treatment plan decisions. As a secondary aim, we also explored whether there were basic professional‐level predictors of decision‐making, such as professional background and years of experience, and whether this was moderated by vignette‐type.

## METHODS

The study was given full ethical approval from the University of Bath Research Ethics Committee and the Health Research Authority.

### Participants

Participants were 270 professionals working in child and adolescent mental health services from a range of different sectors, including the NHS, charity sector, social services, and private practice. Eligibility criteria were (i) any professional involved in mental health diagnosis and/or treatment decision‐making for children and young people; (ii) fluent in English; and (iii) based in the United Kingdom. Most participants (74%; *n* = 201) identified themselves as being from the NHS (*n =* 138 from CAMHS and *n* = 63 from specialist/targeted CAMHS). Participants were recruited via formal and informal pathways to ensure a range of professionals took part. Informal recruitment involved sharing information about the study on social media and via direct email to charity and private sector organizations. We also recruited NHS staff directly via Trusts, following Clinical Research Network and Health Research Authority procedures. In total, 25 trusts across England and Scotland recruited participants for this study.

### Procedure

The survey was administered via Qualtrics. Potential participants accessed an online link, which first took them to informed consent procedures. Participants were told that we were investigating the decision‐making of mental health professionals who work with young people (i.e., there was no mention of PTSD or of the focus on children in care). After providing consent, participants continued to the first part of the survey where they provided basic demographic information and details about their professional qualifications and experience. Participants were then randomly assigned to one of two vignettes describing a 13‐year‐old boy (see Appendix S1). These two vignettes were identical in terms of experience and symptoms. The child was reported to have been exposed to significant violence. The symptoms described in the vignette mimicked common symptoms reported by young people and their caregivers in a study of PTSD in children in care (Hiller et al., 2021). We particularly focused on observable symptoms reported by caregivers, given young people in care may not readily disclose less observable or internalised symptoms (Hiller et al., 2021). Further input was then provided by two specialist PTSD clinicians (including co‐author RMS). In one vignette the child was placed in care (living with a foster carer) and in the other vignette they remained in the care of their biological mother.

#### Diagnosis and treatment decisions

The participants were asked to decide what they thought the primary presenting issue or diagnosis would be, based on 13 possible options. These 13 options were taken from a list of common diagnosable mental health problems in children in care and their peers, based on large‐scale epidemiological studies (e.g., Ford et al., 2007), as well as some other topical and potentially relevant options (e.g., developmental trauma; emerging personality disorder). See Appendix S1 for the list of potential diagnoses. Following the primary diagnosis, participants were then asked to report on what treatment approach they would take, based on a list of 18 therapies. This included NICE recommended treatments, as well as broader but often‐used general therapeutic approaches, such as creative‐based therapies. Finally, they were asked whether there was any other potential secondary diagnosis that they would explore, based on the same list of 13 presenting problems. Following completion of the main components, the study also included qualitative feedback and descriptive information on decision‐making specific to PTSD, which is not presented here.

#### Professional background and experience

In the UK, mental health assessments and treatments can be provided by professionals from a wide variety of backgrounds. Participants were given a list of professions and asked to identify which one they were. These were then collapsed into three categories based on (i) those whose training would likely include significant focus on diagnostic frameworks (i.e., psychiatrists and clinical psychologists); (ii) those trained primarily as psychotherapists and are involved in planning and delivering therapy (e.g., psychotherapists, counselling psychologists, and psychological well‐being practitioners); and (iii) those from broader allied health/social care backgrounds (e.g., mental health nurses, social workers, and occupational therapists). These three profession categories are based on the categorisation used in a similar study with UK‐based clinicians investigating their diagnosis and treatment of panic disorder (Baker & Waite, 2020). Participants also reported on their years of experience in their profession. After completion of the vignette‐related questions, participants were also asked whether they had previously worked with a young person with PTSD (coded as yes/no) and whether they had previously worked with children in care (coded as yes/no).

### Data analysis

Using Statistical Package for the Social Sciences (SPSS), we first conducted chi‐square analyses to investigate the basic associations between vignette type (living with foster carer vs. living with mother) and (i) primary diagnosis of PTSD (yes/no), (ii) a NICE‐recommended PTSD treatment (tf‐CBT or EMDR; yes/no); and (iii) secondary diagnosis of PTSD (yes/no). Whilst it was presumed that a primary diagnosis of PTSD would be predictive of choosing a NICE‐recommended PTSD treatment (tf‐CBT/EMDR), we checked this assumption using descriptive statistics and logistic regression, and also had planned to look at whether this differed by vignette type.

Next, we ran two logistic regressions to investigate whether professional factors predicted (i) whether or not participants chose PTSD as a primary diagnosis and (ii) whether or not participants chose tf‐CBT/EMDR as a treatment (regardless of diagnosis). Predictors were (i) the type of profession (categorical variable: diagnostic frameworks vs. psychological therapies vs. allied health/social care), (ii) years of experience (continuous variable), and (iii) PTSD experience (yes vs. no). Vignette type (living with foster carer vs. mother) was explored as a moderator variable. In the first regression, the outcome variable was whether the clinician suggested PTSD as a primary diagnosis (yes vs. no). In the second regression, the outcome variable was whether the clinician suggested tf‐CBT/EMDR as their likely treatment approach (yes vs. no).

As discussed in detail later, a diagnosis or presenting issue of ‘developmental trauma’ was commonplace. Thus, we also ran further exploratory analyses, to understand what this label meant for decision making. We used descriptive statistics (frequency and percentages) to describe the most common treatment decisions from this label, and ran a single logistic regression to explore whether a diagnosis of developmental trauma (vs. other diagnoses) was predictive of selecting a PTSD treatment (tf‐CBT/EMDR) and whether this differed for vignette type. For this analysis, those who chose PTSD as the primary diagnosis were excluded, to compare a decision of developmental trauma (vs. other diagnoses; excluding PTSD).

### Power analysis

The sample size is based on an a priori power analysis in G*power (Faul et al., 2009). The power was set at 80%, the alpha level at .05, with a binomial X distribution (X parm π = .5) and using medium effect size (OR = .4), based on recent studies investigating the diagnosis and treatment decision‐making of mental health practitioners (e.g., Baker & Waite, 2020; Lee et al., 2017). This showed a required sample of 171 for the logistic regression analyses to have adequate power.

## RESULTS

### Descriptive statistics

See Table 1 for full participant details. Participants were 270 mental health professionals. The majority worked in NHS CAMHS (51%) or specialist NHS CAMHS (23%), and the most common profession was a clinical psychologist (23%). Years of experience ranged from new to the profession (<1 year) to 44 years (*M* = 13.59, SD = 10.26). Most identified that they had previously worked with a young person with PTSD (78.5%) and with a young person in care (83.7%).

**TABLE 1:** Frequencies of key personal and professional characteristics of the mental health professionals that completed the survey (*N* = 270)

Characteristic	% (*n*)
**Gender**	
Female	83.7 (226)
Male	15.6 (42)
Non‐binary	0.4 (1)
**Age**	
18–25	2.6 (7)
26–35	30.7 (83)
36–45	23.3 (63)
46–55	26.3 (71)
56–65	13.3 (36)
66+	3.3 (9)
**Professional background**	
Diagnostic frameworks (e.g., clinical psychologist, psychiatrist)	33.1 (88)
Psychological therapies (e.g., psychotherapist, PWP)	41.4 (110)
Allied health/social care (e.g., mental health nurse)	25.6 (68)
**Current service**	
CAMHS	51.1 (138)
Specialist CAMHS	23.3 (63)
Charity	5.6 (15)
Private/independent practice	5.2 (14)
Social care	0.7 (2)
Other^a^	13.7 (37)

Gender and age information not reported for 1 participant. Current service information missing for 1 other participant. Professional background information missing for 4 participants as they selected ‘other’ and did not go on to clearly describe their roles.

^a^ Large proportion of ‘other’ category worked across multiple services (e.g., NHS and private practice).

### Primary diagnosis

#### Primary diagnosis of PTSD

Across the full sample, 22.7% of participants chose PTSD as the primary diagnosis. There was a significant difference between the proportion of participants who identified PTSD as the primary diagnosis in the foster carer vignette versus the mother vignette (Χ^2^ [1] = 10.09, *p* = .001, OR = 2.65). A primary diagnosis of PTSD was significantly more likely for participants randomized to the mother vignette (31.0%) than the foster carer vignette (14.5%).

#### Common primary diagnosis decisions

The most common primary diagnoses or presenting issues suggested by clinicians who were presented with the mother vignette were developmental trauma (51.2%), PTSD (31.0%), and attachment problems (7.0%). These diagnoses were also most commonly suggested by clinicians presented with the foster carer vignette, but in a different order. For this vignette, developmental trauma remained most common (57.3%), followed by attachment problems (22.1%), and PTSD (14.5%). See Table 2 for a full breakdown of primary diagnoses.

**TABLE 2:** Frequencies of primary and secondary diagnoses given by clinicians when presented with foster carer vignette or mother vignette

	Primary diagnosis		Secondary diagnosis	
	Mother vignette (%) *n* = 129	Foster carer vignette (%) *n* = 131	Mother vignette (%) *n* = 125	Foster carer vignette (%) *n* = 123
Developmental trauma	51.2	57.3	15.2	10.6
PTSD	31.0	14.5	11.2	17.1
Attachment problems	7.0	22.1	31.2	45.5
Major depressive disorder	5.4	3.1	15.2	4.9
ADHD	2.3	0.8	13.6	8.1
Disruptive mood dysregulation disorder	1.6	–	2.4	3.3
Generalized anxiety disorder	0.8	–	4.8	4.9
Emerging antisocial personality disorder	0.8	–	0.8	–
Separation anxiety disorder	–	0.8	0.8	0.8
Social anxiety disorder	–	0.8	3.2	3.3
Oppositional defiant disorder	–	0.8	1.6	1.6

*Note*. For the primary diagnosis, data was missing for 2 clinicians in the mother group and 8 in the foster carer group. For secondary diagnosis, data was missing for 6 clinicians in the mother group and 16 in the foster carer group. No participant selected panic disorder or conduct disorder.

Abbreviations: ADHD, attention deficit hyperactivity disorder; PTSD, Post‐traumatic stress disorder.

#### Predictors of primary PTSD diagnosis decision

We next conducted a logistic regression to investigate whether selecting PTSD as a primary diagnosis was associated with professional factors, with vignette type as the moderator. We found a significant main effect of profession type (*p* = .02), so conducted simple contrasts with diagnostic framework professions as the reference category. There was no significant difference between those typically trained in diagnostic frameworks (e.g., clinical psychologists and psychiatrists) and those trained primarily as therapists (e.g., psychotherapists and counsellors; *p* = .86). However, there was a significant difference in decision‐making between those most likely trained in diagnostic frameworks and allied health professionals (e.g., mental health nurses and social workers), with diagnostically‐trained professionals significantly more likely to select PTSD as a primary diagnosis than allied health/social care professionals (OR = 2.74, 95% CI [1.20, 6.27], *p* = .02). When exploring the moderation, there was no evidence that professional background interacted with vignette type in predicting decision‐making (*p* = .80). There was no significant main effect of years of experience (*p* = .90) or PTSD experience (*p* = .98) on whether or not clinicians selected PTSD as the primary diagnosis, therefore we did not go further to investigate potential interaction effects between these professional factors and vignette type. See Table 3 for regression summaries.

**TABLE 3:** Regression summaries of whether professional factors predict diagnosis and treatment decisions made by clinicians

	χ^2^	*Df*	*Wald*	B	SE	OR
PTSD primary as dependent variable	19.26*	8				
Profession type			7.44*			
*Diagnostic* vs. *Therapist*			.03	0.08	.43	1.08
*Diagnostic* vs. *Allied*			5.69*	1.01	.42	2.74
Years of experience			0.01	−0.00	.02	0.99
PTSD experience			0.00	−0.01	.44	0.99
PTSD secondary as dependent variable	5.20	8				
Profession type			0.65			
Years of experience			0.05	0.01	.02	1.01
PTSD experience			0.03	−0.09	.52	0.92
TF‐CBT as dependent variable	12.30	8				
Profession type			4.86			
Years of experience			0.03	−0.00	.02	0.99
PTSD experience			1.00	−0.45	.44	0.64
EMDR as dependent variable	1.68	8				
Profession type			0.12			
Years of experience			0.10	0.01	.03	1.01
PTSD experience			0.83	0.54	.59	1.71

Abbreviations: EMDR, eye movement desensitization and reprocessing; PTSD, Post‐traumatic stress disorder; TF‐CBT, trauma‐focused cognitive behavioural therapy.

*p < .05; **p < .01; ***p < .001.

### Treatment

#### TF‐CBT/EMDR

Overall, 33.9% of the sample chose a NICE‐recommended PTSD treatment (tf‐CBT or EMDR). We found a significant difference between vignettes for the proportion of participants who selected tf‐CBT or EMDR as the treatment approach (Χ^2^ [1] = 4.94, *p* = .03, OR = 1.82). Participants were more likely to select either tf‐CBT or EMDR if they were randomized to the mother vignette (40.4%) than the foster carer vignette (27.2%).

#### Common treatment decisions

The most common types of treatment suggested by clinicians presented with the mother vignette were tf‐CBT (34.1%), systemic therapy (15.1%), and CBT (8.7%). Clinicians presented with the foster carer vignette also suggested tf‐CBT most frequently (19.2%). However, this was followed by child and adolescent psychotherapy (16.8%) and dyadic developmental psychotherapy (14.4%) as the next most frequent treatment suggestions for this vignette. See Table 4 for the full breakdown of treatment decisions.

**TABLE 4:** Frequencies of treatment type suggested by clinicians when presented with foster carer vignette or mother vignette

Treatment	Mother vignette (%) *n* = 126	Foster carer vignette (%) *n* = 125
Trauma‐focused CBT	34.1	19.2
Systemic therapy	15.1	8.8
CBT	8.7	7.2
EMDR	6.3	8.0
Child and adolescent psychotherapy	6.3	16.8
Dyadic developmental psychotherapy	5.6	14.4
Parent/caregiver training programme	5.6	5.6
Family therapy	4.8	0.8
Dialectical behaviour therapy	4.0	1.6
Art or creative based therapy	3.2	5.6
Behaviour activation	3.2	–
Play therapy	0.8	5.6
Psychoanalytic psychotherapy	0.8	1.6
Brief solution‐focused therapy	0.8	1.6
Behaviour management programme	0.8	–
Mentalisation‐based therapy	–	1.6

CBT, Cognitive behavioural therapy; EMDR, Eye movement desensitization and reprocessing. Data were missing for 5 clinicians in the mother group and 15 in the foster carer group. No participant selected mindfulness‐based therapy or hynotherapy.

#### Predictors of tf‐CBT/EMDR treatment decision

Next, we used logistic regression to investigate whether professional factors influenced whether clinicians chose tf‐CBT/EMDR (i.e., a NICE‐recommended PTSD treatment) versus any other treatment. We found that there were no significant main effects of profession type (*p* = .15), years of experience (*p* = .91), or PTSD experience (*p* = .71), therefore we did not go further to investigate potential interaction effects between professional factors and vignette type on treatment decision (see Table 3).

### Secondary diagnosis

#### Secondary diagnosis of PTSD

Here, we found that there was no significant association between vignette type and secondary PTSD diagnosis (Χ^2^ [1] = 1.76, *p* = .18), meaning there was no difference between responses from participants presented with the mother versus foster carer vignette (see Table 2).

#### Common secondary diagnosis decisions

Participants randomised to the mother vignette selected attachment problems as the highest secondary diagnosis (31.2%), then developmental trauma and major depressive disorder were next highest (both 15.2%), closely followed by ADHD (13.6%). Almost half of the clinicians who were presented with the foster carer vignette also suggested attachment problems as a secondary diagnosis (45.5%), followed by PTSD (17.1%) and developmental trauma (10.6%; Table 2).

### Associations between diagnosis and treatment

#### Post‐traumatic stress disorder

For the overall sample, of those who selected PTSD as the primary diagnosis, 52.7% then selected a NICE‐recommended PTSD treatment. For those randomized to the mother vignette, where PTSD was chosen as a primary diagnosis (*n* = 40), the most common treatments were tf‐CBT (38.5%), CBT (20.5%), and EMDR (10.3%). For those randomized to the foster carer vignette, where PTSD was chosen as the primary diagnosis (*n* = 19), the most common treatments were tf‐CBT (37.5%), EMDR (25.0%), and CBT (12.5%). Thus, only 48.8% of those randomized to the mother vignette who selected PTSD as a primary diagnosis suggested a NICE recommended PTSD treatment, and 62.5% of those randomized to the foster carer vignette who selected PTSD as a primary diagnosis suggested a NICE recommended PTSD treatment.

As expected, a primary diagnosis of PTSD (vs. any other diagnosis) significantly predicted an increased likelihood of selecting a PTSD treatment (tf‐CBT or EMDR); across all participants, professionals who chose PTSD as the primary problem were three times more likely to select a NICE‐recommended PTSD treatment (Wald = 10.61, B = 1.02, SE = .31, OR = 3.21, 95% CI [1.50, 5.14], *p* = .001). We did not go further to investigate whether there was an interaction effect of vignette type, as the sub‐sample of those selecting PTSD as a primary diagnosis was too small.

#### Developmental trauma

In both vignettes, a diagnosis of developmental trauma was by far the most common choice (just over 50% in both cases). Therefore, we decided post‐hoc to explore treatment decisions based on this diagnosis/presenting problem. For those randomized to the mother vignette, where a primary diagnosis/presenting problem of developmental trauma was chosen (*n* = 66), the most common treatment decisions were tf‐CBT (34.4%), systemic therapy (15.6%), and child and adolescent psychotherapy (10.9%). For those randomized to the foster carer vignette (*n* = 75), the most common treatment decisions were child and adolescent psychotherapy (21.4%), dyadic developmental psychotherapy (21.4%), and tf‐CBT (15.7%). See Table 5 for the full list of treatment decisions.

**TABLE 5:** Frequencies of treatment type suggested by clinicians who gave either post‐traumatic stress disorder (PTSD) or developmental trauma as their primary diagnosis

	PTSD % (*n* = 55)	Developmental trauma % (*n* = 134)
Trauma‐focused CBT	38.2	24.6
CBT	18.2	2.2
EMDR	14.5	7.5
Systemic therapy	10.9	11.9
Family therapy	3.6	3.0
Art/Creative‐based therapy	3.6	4.5
Child and adolescent psychotherapy	3.6	16.4
Play therapy	1.8	2.2
Psychoanalytic psychotherapy	1.8	0.7
Behaviour activation	1.8	–
Dialectical behaviour therapy	1.8	2.2
Dyadic developmental psychotherapy	–	15.7
Parent/caregiver training programme	–	6.7
Brief solution focused therapy	–	1.5
Mentalization‐based therapy	–	0.7

Abbreviations: CBT, Cognitive behavioural therapy; EMDR, eye movement and desensitization and reprocessing.

Finally, we conducted a logistic regression (with primary PTSD cases removed so as not to skew findings) and found no significant main effect of developmental trauma in predicting whether or not the professional chose tf‐CBT/EMDR (*N* = 192, Wald = 3.04, B = .66, SE = .38, OR = 1.93, 95% CI [.92, 4.06], *p* = .08). Thus, overall a primary decision of developmental trauma did not predict an increased likelihood of selecting a NICE‐recommended PTSD treatment. As this was not significant, we did not go on to investigate potential interaction effects between primary developmental trauma and vignette type.

## DISCUSSION

We used an experimental vignette design to explore whether a young person being in care might influence clinical diagnosis and treatment decision‐making, specific to PTSD. When presented with identical history and symptom summaries, mental health professionals randomized to the foster carer vignette were significantly less likely to give a primary diagnosis of PTSD and select a NICE recommended PTSD treatment (tf‐CBT or EMDR). As expected, choosing PTSD as a primary diagnosis meant the clinician was more likely to go on to choose tf‐CBT or EMDR, highlighting the importance of diagnoses in influencing treatment decision‐making.

There is evidence that PTSD is often under‐assessed and diagnosed in child welfare settings (Grasso et al., 2009; Miele & O'Brien, 2010; Morris et al., 2015). Here, we extend these findings by directly comparing decisions for young people not in care using case‐based vignettes. Our findings show a potential diagnosis and treatment decision bias towards children in care versus their peers, even when the presenting symptoms are the same. This has important clinical and training implications. Given most of our sample had experience working with children in care and with children with PTSD, yet the diagnosis and treatment bias remained, our findings suggest training for mental health professionals is crucial to specifically address potential assumptions or biases that may act as barriers to children in care receiving appropriate diagnoses and thus treatments. Children in care are 12 times more likely to meet PTSD criteria than their peers (Ford et al., 2007). Therefore, the lesser detection here, where there arguably should be the assumption of higher rates, is particularly noteworthy.

Overall, the identification of PTSD as the primary issue was relatively low across the full sample (23%). Of course, in real‐world practice, a professional would likely gather far more detailed information than provided in this vignette, which may then increase the identification of PTSD. Our vignettes were not designed to capture all symptoms of PTSD, and some symptoms described might overlap with other diagnoses. Whilst a PTSD diagnosis increased the likelihood of a professional choosing tf‐CBT or EMDR as the treatment, this was still only the case for 53% of professionals who selected PTSD as the primary diagnosis. Thus, around half of professionals who chose PTSD as the diagnosis still did not then choose a NICE‐recommended PTSD treatment. We know that lack of access to training and supervision is a key barrier to the use of trauma‐focused CBT (Finch et al., 2020). Strategies to feasibly increase knowledge and skills in, as well as the implementation of PTSD‐specific interventions within existing services, remains a crucial area of investigation. Of course, training alone may not be enough, and research is needed to continue to investigate the layers of barriers that may exist in supporting services to use these types of treatments with children in care (and young people in general).

Whilst PTSD was lesser diagnosed, trauma‐related distress was clearly acknowledged via the frequent choice of ‘developmental trauma’ as the primary diagnosis/presenting problem. While developmental trauma was the most commonly chosen diagnosis or presenting issue, this is not a formal diagnosis in either the DSM‐5 (American Psychiatric Association, 2013) or ICD‐11. Efforts were made to introduce developmental trauma disorder as a formal diagnosis in the last DSM revisions, but it was ultimately not included – although complex PTSD was included in ICD‐11, which has a similar symptom presentation (McFetridge et al., 2017). There remains significant debate about developmental trauma disorder and its inclusion as a formal diagnosis. It was ultimately not included within existing diagnostic systems, mainly as the criteria overlaps with many other established diagnoses (e.g., borderline personality disorder, attachment disorders, and conduct disorder; Schmid et al., 2013). There is also concern about conflating the experience of trauma with the mental health outcome (i.e., developmental trauma is often the term used for *exposure* to maltreatment), given not all children exposed to maltreatment will develop mental health difficulties. Whilst there is no specific treatment guidance for ‘developmental trauma’ as a diagnosis/presenting problem, we found that 40% of professionals who were randomized to the mother vignette, still chose a NICE‐recommended PTSD treatment. This was slightly less common (24%) for professionals randomized to the foster carer vignette. Here, developmental trauma led to various different treatment approaches, including DDP and general psychotherapy – neither of which are currently evidence‐based treatments for PTSD. Indeed, there remains no randomized controlled trial of DDP, despite its common use in practice (although one is currently underway; Turner‐Halliday et al., 2014). Our findings support research suggesting that children in care may be less likely to access existing best‐evidenced interventions (Blower et al., 2004; Morris et al., 2015).

Beyond developmental trauma, over one in five (22%) professionals who were randomized to the foster carer vignette chose attachment problems as the primary diagnosis/presenting problem. This was the case in 7% (less than one in 10) of those randomized to the mother vignette. Though it is presumed attachment difficulties are more prevalent for children in care due to their early experiences, there has been much debate about both the over and under‐diagnosis of attachment problems for this group. While the vignette made mention of difficulties in relationships, it did not include common attachment disorder related symptoms, such as lack of remorse, attention seeking, or inappropriate comfort seeking/boundaries (Lehmann et al., 2020). Findings provide some initial quantitative evidence supporting concerns that attachment disorders are used as a ‘catch all’ for children in care, even when presentations do not match diagnostic systems, which may mean more common problems are overlooked (Woolgar & Scott, 2014).

This study has various strengths, including the large sample of mental health professionals from a variety of professional backgrounds, reflecting the types of professions and sectors that provide mental health support to children in the UK. Another strength is the experimental methods used to investigate and quantify biases in diagnosis and treatment decisions. However, findings should be considered in light of various caveats. First, as already highlighted, the vignettes provided only basic information. In a real clinical setting, mental health professionals would likely collect more details of the young person's experiences and symptoms or difficulties. That said, it is also the case that triaging decisions (for what services might be made available) are often made on very limited information. Triaging decisions may be particularly important in relation to PTSD diagnoses, given our finding that participants from professions typically trained in diagnostic frameworks (e.g., clinical psychologists) were more likely to select a PTSD diagnosis than those from broader allied health backgrounds (e.g., mental health nurses). Of course, such findings are unsurprising, and may be related to differences between these professions in their confidence in recognizing and treating trauma (Finch, 2019). However, this means that if PTSD is not identified as a potential diagnosis at triaging stage, a young person may be triaged into a service where diagnoses are not routinely made and PTSD treatments not offered. Relatedly, the use of an experimental vignette also required forced reporting on diagnoses and treatment decisions. We know that many mental health workers may have found that very artificial from their usual practice. However, this was necessary to explore differences between the vignettes, which was the focus here. There also may have been other diagnosis and treatment options that were not on the list, which they would have chosen, although the focus of this work was on PTSD. Finally, findings cannot be generalized to diagnoses other than PTSD, but we hope might prompt further exploration of diagnosis and treatment decision‐making for young people in care.

This study has presented quantifiable evidence of a diagnostic and treatment decision‐making bias faced by children in care, and wider issues around the recognition and management of PTSD in children and young people across all settings. This bias, at least in part, potentially reflects the ongoing debate around the use of diagnostic frameworks for children exposed to ongoing childhood trauma, and particularly those in care. While such debate is important for the advancement of research and practice, the current study showed that the diagnosis of PTSD was important for the flow‐on treatment decision and therefore the use of best‐evidenced treatments, in particular for children in care. Mental health professionals face difficult decisions in diagnoses and treatments, particularly when faced with other complexities or acute crises and within under‐resourced systems (Finch et al., 2020). Nevertheless, it remains crucial to explore feasible and scalable ways to increase the appropriate detection of diagnosable mental health disorders, to support access to psychoeducation and best‐evidenced treatments.

## AUTHOR CONTRIBUTION

RM: Co‐conceptualisation; led the data collection, data analysis and writing of manuscript. SH: Involved in the conceptualisation of the project, co‐supervision of RM, and input on analysis and write‐up. RM‐S: Involved in conceptualisation of the project, development of vignettes, feedback on analysis, and write‐up. LD: Supported development of survey and data collection; feedback on manuscript. RH: Co‐led the conceptualisation of the project; primary supervision of RM; manuscript development and final approval.

## CONFLICT OF INTEREST

All authors declare no conflict of interest.

## Data Availability

The data that support the findings of this study are available from the corresponding author upon reasonable request.
