# Supplementary material for: Differences in the diagnosis and treatment decisions for children in care compared to their peers: An experimental study on post‐traumatic stress disorder
Source: Br J Clin Psychol. 2022 Jun 14;61(4):1075–88. doi: 10.1111/bjc.12379 (PMC9796033; doi:10.1111/bjc.12379)
Supplement: Supplementary file 1 [file BJC-61-1075-s001.docx]

**Supplementary material**

*Vignette (Only 1 is displayed):*

Please read the vignette below and answer some questions about it.

**Care-experienced:** Conor is a 13 year old boy. Until he was 10 years old he lived with his mother and father. There was a lot of violence in the home. At 10 years old he was placed in the care system and Connor now lives with a foster carer, whom he has lived with for the past two and a half years. He is now physically safe. Connor is physically healthy with no developmental difficulties.

Connor’s foster carer describes him as difficult to get along with. He rarely seems happy and is quick to anger, sometimes for no apparent reason at all, and can take hours to calm down. He spends long periods of time in his bedroom. His teachers often comment that he struggles to stay on task or concentrate on his work. He also struggles to make friends. His carer is also concerned that his sleep is very poor as he is very tired during the day. Connor does not like to be asked about what happened in his family home and rarely speaks about it. If he is asked about it he gets a look of ‘freezing up’ and will often leave the house angry or upset. She reports that these have been issues for Connor for the past few years, but seem to be getting worse over the last year.

Connor agrees that he struggles with feelings of anger. He thinks people are mostly out to get him and can’t be trusted. He agrees that he doesn’t have many friends at school but isn’t really interested in making them. He doesn’t like school but says he’s not good at it anyway so it doesn’t matter.

**Not care-experienced:** Conor is a 13 year old boy. Until he was 10 years old he lived with his mother and father. There was a lot of violence in the home. At 10 years old Connor and his mother moved away from his father and since then it has just been the two of them. He is now physically safe. Connor is physically healthy with no developmental difficulties.

Connor’s mum describes him as difficult to get along with. He rarely seems happy and is quick to anger, sometimes for no apparent reason at all, and can take hours to calm down. He spends long periods of time in his bedroom. His teachers often comment that he struggles to stay on task or concentrate on his work. He also struggles to make friends. His mum is also concerned that his sleep is very poor as he is very tired during the day. Connor does not like to be asked about what happened when his dad was around and rarely speaks about it. If he is asked about it he gets a look of ‘freezing up’ and will often leave the house angry or upset. She reports that these have been issues for Connor for the past few years, but seem to be getting worse over the last year.

Connor agrees that he struggles with feelings of anger. He thinks people are mostly out to get him and can’t be trusted. He agrees that he doesn’t have many friends at school but isn’t really interested in making them. He doesn’t like school but says he’s not good at it anyway so it doesn’t matter.

*Diagnosis options listed:*

Attachment problems  Major Depressive Disorder

Attention Deficit Hyperactivity Disorder  Oppositional Defiant Disorder

Conduct Disorder  Panic Disorder

Developmental trauma  Posttraumatic Stress Disorder

Disruptive Mood Dysregulation Disorder  Separation Anxiety Disorder

Emerging Antisocial Personality Disorder  Social Anxiety Disorder

Generalised Anxiety Disorder

*Treatment options listed:*

Art or creative-based Therapy  Family therapy

Play therapy  Systemic therapy

Behaviour activation  Parent/caregiver training programme

Behaviour management programme  Child and Adolescent Psychotherapy

Dialectical behaviour therapy  Cognitive behavioural therapy

Hypnotherapy  Trauma-Focused CBT

Brief Solution Focused Therapy  Mentalization-based therapy

Psychoanalytic Psychotherapy  Mindfulness-based therapy

Dyadic Developmental Psychotherapy  Eye Movement Desensitization and Reprocessing
